# Left atrial strain predicts the rhythm outcome in patients with persistent atrial fibrillation undergoing left atrial cryoablation during minimally invasive mitral valve repair

**DOI:** 10.3389/fcvm.2024.1373310

**Published:** 2024-03-27

**Authors:** Yalin Yildirim, Sevenai Yildirim, Johannes Petersen, Yousuf Alassar, Harun Sarwari, Christoph Sinning, Stefan Blankenberg, Hermann Reichenspurner, Simon Pecha

**Affiliations:** ^1^ Department of Cardiovascular Surgery, University Heart and Vascular Center Hamburg, University Medical Center Hamburg Eppendorf, Hamburg, Germany; ^2^ Department of Cardiology, University Heart and Vascular Center Hamburg, University Medical Center Hamburg Eppendorf, Hamburg, Germany; ^3^ German Center for Cardiovascular Research (DZHK) Partner Site Hamburg/Kiel/Lübeck, Hamburg, Germany

**Keywords:** atrial fibrillation, surgical ablation, arrhythmia surgery, left atrial strain, AF surgery

## Abstract

**Objectives:**

Patients with atrial fibrillation (AF) have lower left atrial (LA) strain, which is a predictor for LA function. Here, we evaluated the prognostic value of LA strain to predict the rhythm outcome in patients with persistent AF undergoing LA cryoablation concomitant to minimally invasive mitral valve repair.

**Methods:**

Between 01/2016 and 12/2020, 72 patients with persistent AF underwent LA cryoablation during minimally invasive mitral valve surgery. All patients received a complete LA lesion set and left atrial appendage (LAA) closure with a clip. All patients received preoperative transthoracic echocardiography (TTE) with LA and left ventricular strain measurements. Preoperative LA and LV strain analysis was correlated with postoperative rhythm outcome.

**Results:**

The mean age of the patients was 66.9 ± 7.2 years, of whom 42 (58%) were male patients. No major ablation-related complications occurred in any of the patients. Successful LAA closure was confirmed by intraoperative echocardiography in all patients. The 1-year survival rate was 97%. Freedom from AF at 12 months was 72% and 68% off antiarrhythmic drugs. Preoperative LA strain values were statistically significantly higher in patients with freedom from AF at 12 months of follow-up (12.7% ± 6.9% vs. 4.9% ± 4.1%, *p* = 0.006). Preoperative LV strain value was not associated with postoperative rhythm outcome. In multivariate logistic regression analysis, LA strain (*p* < 0.001) and AF duration (*p* = 0.017) were predictors for freedom from AF at 12 months of follow-up.

**Conclusions:**

In our study, LA strain analysis predicted the rhythm outcome in patients with persistent AF undergoing concomitant surgical AF ablation. In the future, LA strain might be a useful tool to guide decision-making on ablation strategies in patients with persistent AF.

## Introduction

Atrial fibrillation (AF) is the most common arrhythmia in the Western world. It is associated with an increased number of thromboembolic events, including stroke, and an increased mortality rate (1–4). Furthermore, it leads to heart failure and contributes to an increased number of hospitalizations (1–4). Concomitant surgical AF ablation has been shown to reduce AF in retrospective and prospective randomized trials (5–10). In prospective registries and retrospective studies, a survival benefit has been shown for patients with AF undergoing concomitant AF surgery (7, 11–13). Therefore, concomitant surgical AF ablation is recommended in the recent guidelines of the European Society of Cardiology, European Heart Rhythm Society/Heart Rhythm Society and Society of Thoracic Surgeons (14–16).

The initial cut-and-sew Cox-Maze procedure has been modified to the Cox-Maze III procedure, which has been the gold standard for many years (17). However, due to the complexity of this technique, it has only been used by a small group of surgeons. Replacing the cut-and-sew technique with the creation of thermal lesions has simplified the procedure and resulted in a widespread application as the Cox-Maze IV procedure. Over the years, many modifications of the original Cox-Maze IV lesion set have been used. Various lesion sets with varying results have been published in recent years and there is no clear evidence of which lesion set to use in which patient. Especially in patients with long-standing persistent AF, many surgeons are reluctant to perform any kind of surgical AF ablation.

Left atrial (LA) strain is a parameter that can be easily obtained using transthoracic echocardiography (18–20). It is a predictor for LA function and is reduced in patients with AF (18–20). We here investigated if preoperative LA strain can be used as a rhythm outcome predictor after LA cryoablation in patients with persistent or long-standing persistent AF.

## Materials and methods

**Figure 1:**
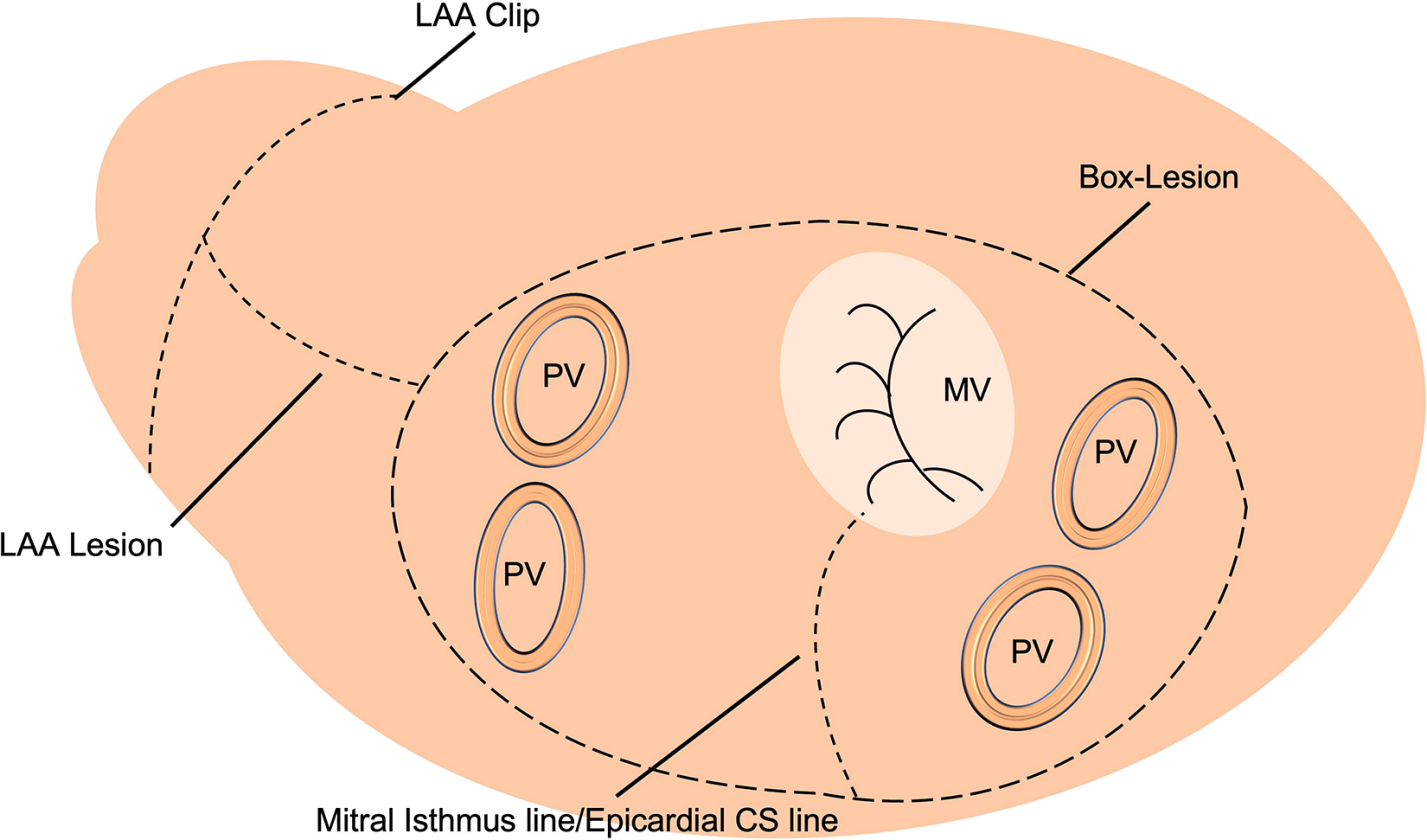
Schematic left atrial (LA) lesion set for cryoablation.

Between 01/2016 and 12/2020, 72 patients with persistent or long-standing persistent AF underwent LA cryoablation during minimally invasive mitral valve surgery and were included in this retrospective data analysis. The institutional review board (IRB) approval (Ethikkomission Ärztekammer Hamburg 2020-10183-BO-ff) was obtained, and patients gave written informed consent. All patients underwent minimally invasive endoscopic mitral valve surgery via right anterolateral thoracotomy. A total of 18 (25%) patients received additional tricuspid valve repair (TVR). The identical LA lesion set including a box lesion and LA appendage and mitral isthmus isolation (endocardial and epicardial) was performed in all patients (Figure 1). A standardized cryoablation protocol was used and cryoenergy was applied for 120 s for each line, at the arrested heart. Furthermore, left atrial appendage (LAA) closure, using the AtriClip (AtriCure Inc. West Chester, Ohio) was conducted in all patients. Applied energy sources included nitrous oxide or argon-based cryoablation (cryoICE Cryoablation Probe, AtriCure Inc., West Chester, Ohio; Cardioblate CryoFlex, Medtronic, Minneapolis, Minnesota). All patients received preoperative transthoracic echocardiography (TTE) with LA and left ventricular strain measurements. Preoperative LA and LV strain analysis was correlated with postoperative rhythm outcome.

### Echocardiography

**Figure 2:**
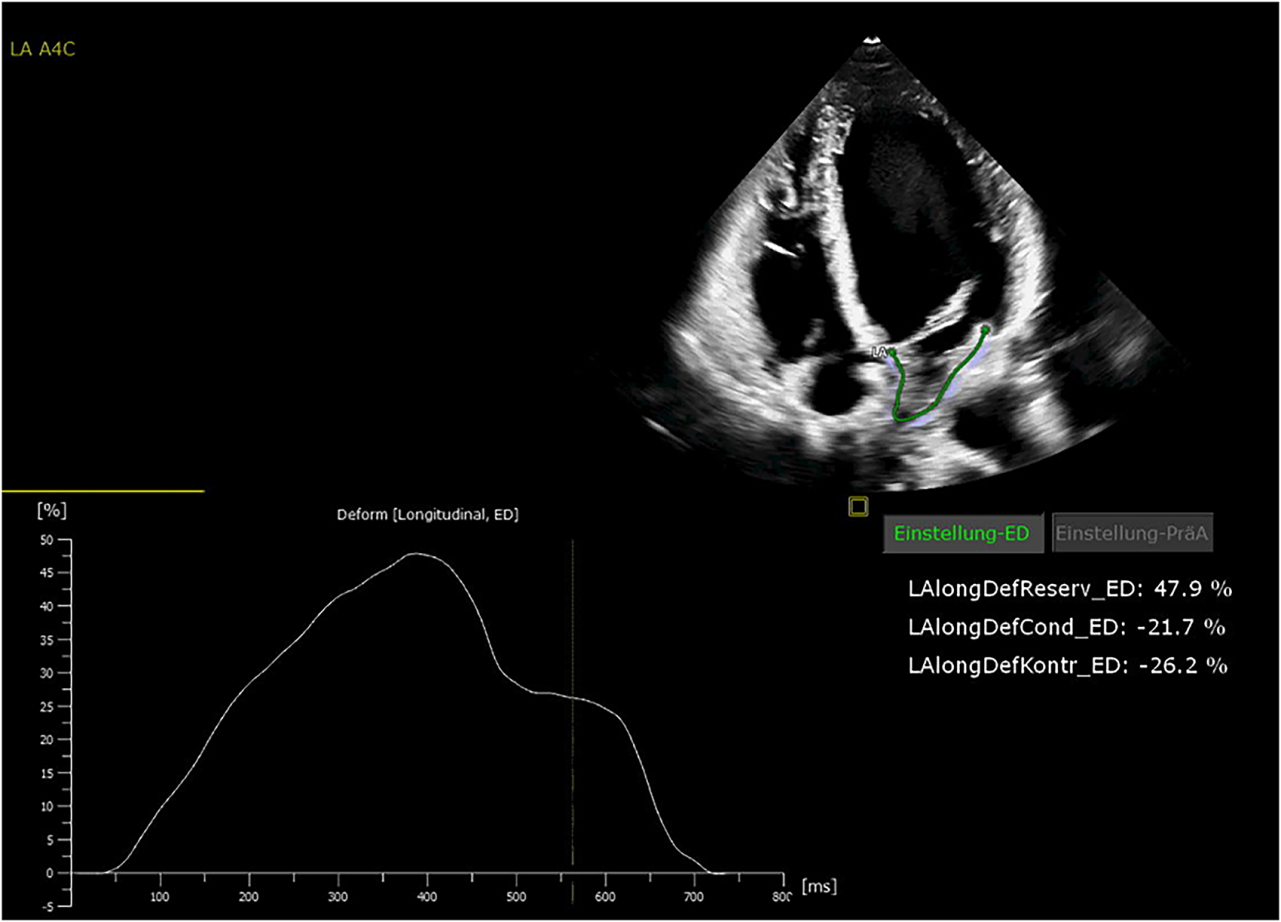
Echocardiography showing normal left atrial (LA) strain values.

**Figure 3:**
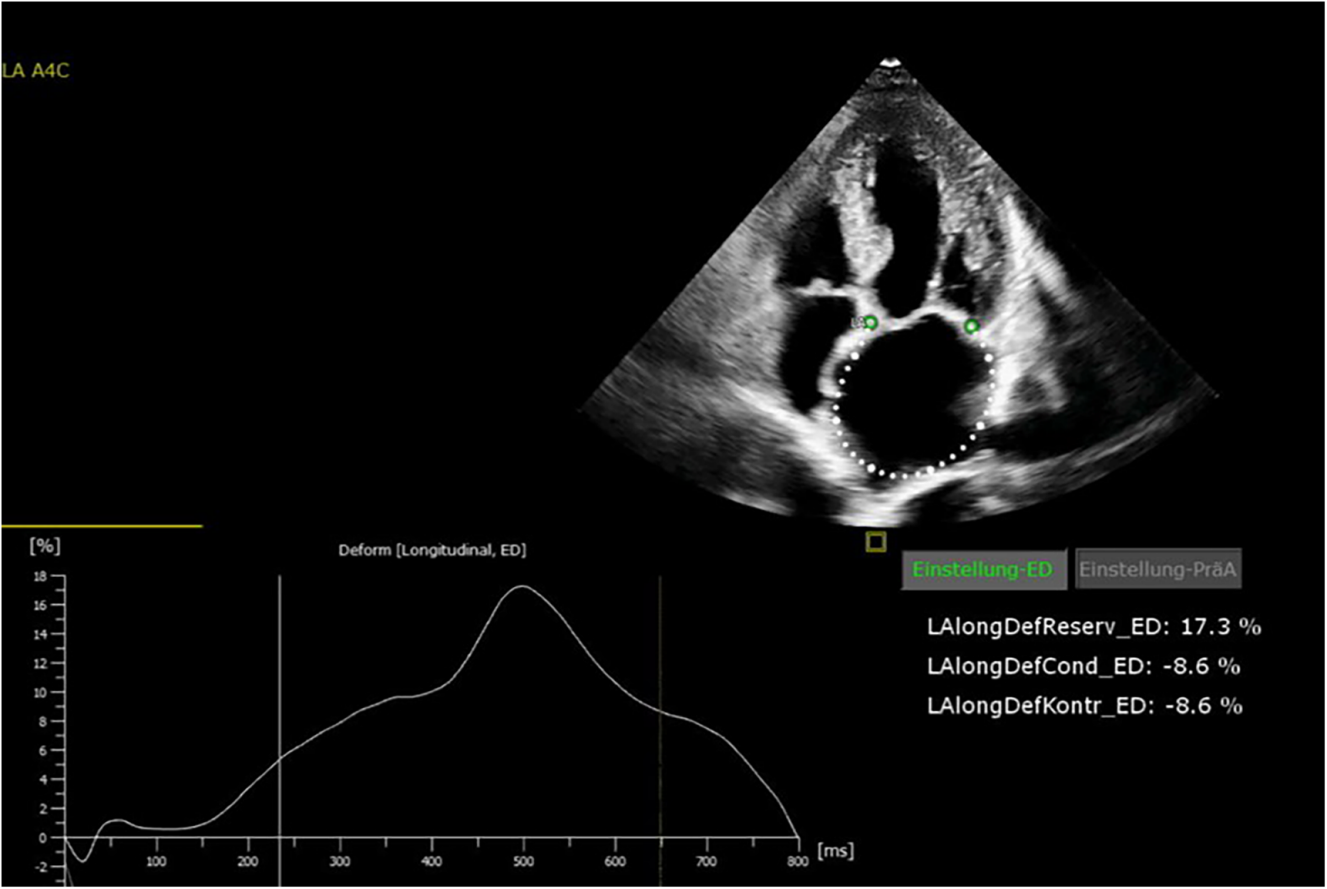
Echocardiography showing reduced left atrial (LA) strain values.

All patients were in AF at the time of preoperative transthoracic echocardiography. The patients were examined by transthoracic echocardiography. After manual tracing of the LA endocardial border, the software Cardiac Performance Analysis (IMAGE-COM, TOMTEC-ARENA, TOMTEC Imaging System GmbH, Unterschleissheim, Germany) automatically tracked the myocardium throughout the cardiac cycle (Figures 2, 3). LA reservoir strain was presented. The same measurements were performed to assess LV strain measurements. The additional echocardiography included an assessment of left ventricular ejection fraction (LVEF), diastolic function, LV hypertrophy, and measurement of LA and left ventricular diameters and volumes. The measurements were according to the current recommendations (21), which are also applicable to strain measurements (22).

### Follow-up

Rhythm follow-up was conducted by 24 h Holter electrocardiogram (ECG) 12 months postoperative. AF recurrence was defined by a single AF episode > 30 sec in 24 h Holter ECG. Antiarrhythmic drugs (AAD) and anticoagulation regimen were maintained for 3 months postoperative in all patients and then adapted according to either CHA_2_DS_2_-VASc score. In patients without contraindications, amiodarone was used as first-line antiarrhythmic drug therapy; otherwise, other class I or III AAD were used for at least 3 months postoperative.

### Statistical analysis

All statistical analyses were performed with SPSS statistical software version 27.0 (SPSS Inc., Chicago, Illinois). Continuous values are expressed as mean ± standard deviation and were compared with the Student’s *t*-test if appropriate; otherwise, the Mann–Whitney *U* test was used. Categorical variables are displayed as frequency and percentages and were compared using the chi-square test or Fisher’s exact test as appropriate. Uni- and multivariate logistic regression analysis was used to identify predictors for freedom from AF at 12 months of follow-up. A *p*-value of <0.05 was considered statistically significant.

## Results

### Patient characteristics

The mean age of the patients was 66.9 ± 7.2 years, of whom 58% were male. All patients presented with persistent- or long-standing persistent AF. The mean LA volume was 96.7 ± 25.5 ml. The mean LVEF was 56.1 ± 8.7%. The mean duration of AF was 3.9 ± 2.7 years. A total of 49 (68.1%) patients had arterial hypertension, and 15 (20.8%) patients had coronary artery disease. Seven (9.7%) patients suffered from chronic kidney disease, and 10 (13.8%) had diabetes mellitus. The detailed patient characteristics are displayed in Table 1.

**Table 1:** Patient baseline characteristics.

	*n* = 72
Age (years)	66.9 ± 7.2
Sex [male *n*, (%)]	42 (58.3)
LA volume (ml)	96.7 ± 25.5
AF duration (years)	3.9 ± 2.7
LVEF (%)	56.1 ± 8.7
Diabetes mellitus *n* (%)	10 (13.8)
Hyperlipidemia	12 (16.7)
Arterial hypertension *n* (%)	49 (68.1)
Chronic kidney disease *n* (%)	7 (9.7)
Coronary artery disease	15 (20.8%)
History of stroke *n* (%)	5 (6.9)
Preoperative pacemaker	3 (4.1%)

AF, atrial fibrillation; LA, left atrial; LVEF, left ventricular ejection fraction.

### Procedural data

The performed surgical procedure was an isolated minimally invasive endoscopic mitral valve repair in 52 patients, an isolated mitral valve replacement in 2 patients, and a combined minimally invasive mitral and TVR in 18 patients. LA cryoablation including box lesion, mitral isthmus lesion, and LAA occlusion using the AtriClip device was performed in all participants. Successful intraoperative LAA closure was confirmed in all patients using transesophageal echocardiography.

The mean cardiopulmonary bypass time was 164.6 min ± 3.3, and the mean cross-clamp time was 87.5 ± 29.3 min. No major ablation-related complication occurred in any of the patients. There was no intra- or perioperative death. The 1-year survival rate was 97.2%. One patient had a (1.4%) stroke during follow-up. Three patients required postoperative pacemaker implantation (4.2%), and two patients (2.8%) received permanent pacemaker implantation during 12 months of follow-up. The detailed procedural data are displayed in Table 2.

**Table 2:** Perioperative data.

	*n* = 72
Isolated mitral valve repair *n* (%)	52 (72.2)
Isolated mitral valve replacement *n* (%)	2 (2.8)
Combined mitral and tricuspid repair *n* (%)	18 (25.0)
Mean cross-clamp time (min)	164.6 min ± 3.3
Mean cardiopulmonary bypass time (min)	87.5 ± 29.3
Successful LAA closure (TEE) *n* (%)	72 (100)
Re-thoracotomy for bleeding *n* (%)	3 (4.2)
In-hospital mortality *n* (%)	0 (0)
Perioperative stroke *n* (%)	0 (0)
Postoperative pacemaker implantation *n* (%)	3 (4.2)

TEE, transesophageal echocardiography; LAA, left atrial appendage; min, minute.

### Rhythm results

**Figure 4:**
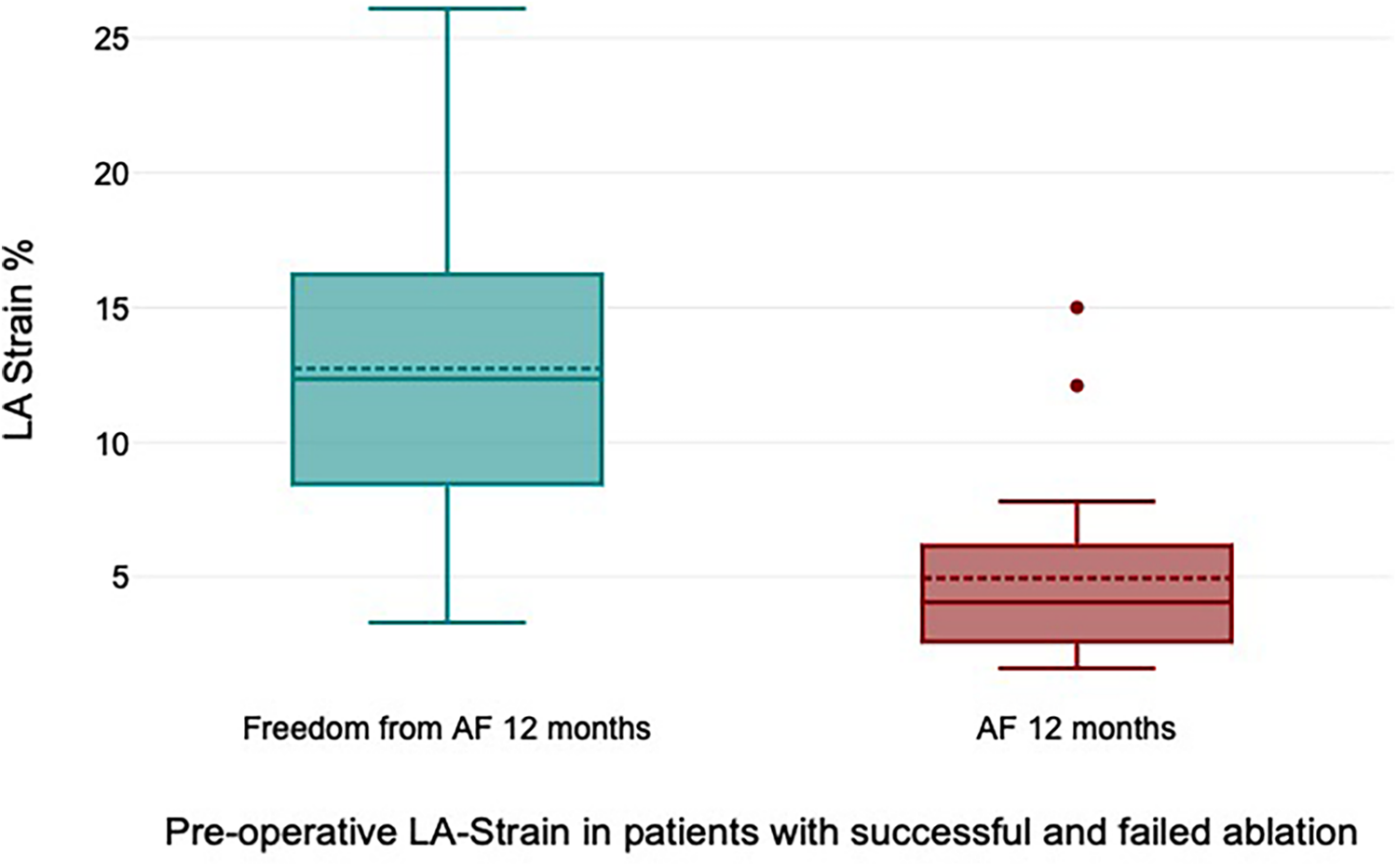
Preoperative left atrial (LA) strain values in patients with successful and failed ablation.

All patients received 24 h Holter ECG at 12 months of follow-up. Freedom from AF was 72% at 12 months of follow-up. Freedom from AF off AAD at 12 months was 68%. The preoperative LA strain value was a statistically significant rhythm outcome predictor at 12 months of follow-up, with significantly higher LA strain values in patients with successful rhythm outcome (12.7% ± 6.9% vs. 4.9% ± 4.1%, *p* = 0.006) (Figure 4). The mean preoperative LA volume was 94.6 ml ± 15.1 ml in patients with successful ablation and 102.4 ± 13.1 ml in patients with failed ablation (*p* = 0.25).

The preoperative LV strain was −12.4% ± 6.1% in patients with freedom from AF at 12 months and −9.4% ± 5.5% in patients with AF during follow-up, showing no statistically significant difference between both groups (*p* = 0.392).

The multivariate logistic regression analysis revealed that LA strain (*p* < 0.001; OR: 3.71; 95% CI 1.29–12.65) and AF duration (*p* = 0.017; OR 2.01; 95% CI 1.33–5.19) were statistically significant predictors for freedom from AF at 12 months, whereas LA volume (*p* = 0.344), sex (*p* = 0.486), age (*p* = 0.166), coronary artery disease (*p* = 0.836), and additional TVR (*p* = 0.195) did not reach statistical significance (Table 3).

**Table 3:** Multivariate logistic regression—predictors for freedom from AF.

Variables	Odds ratio	95% CI	*p*-value
LA strain	3.71	1.29–12.65	*<0.001*
AF duration	2.01	1.33–5.19	*0*.*017*
LA volume	1.42	0.88–6.92	0.344
TVR	3.11	1.31–11.4	0.195
Diabetes mellitus	1.29	0.74–2.01	0.418
Female sex	1.60	0.95–5.17	0.556
LVEF	2.10	0.61–4.36	0.548
Coronary artery disease	1.71	0.90–4.22	0.836
Age	1.61	0.54–5.83	0.934

AF, atrial fibrillation; LA, left atrial; TVR, tricuspid valve repair; LVEF, left ventricular ejection fraction.

Italic values are statistically significant *p*-values.

## Discussion

We analyzed the predictive value of preoperative LA strain in patients with persistent and long-standing persistent AF undergoing cryoablation during minimally invasive mitral valve surgery. In patients with successful rhythm outcome at 12 months, preoperative LA strain was significantly higher than in patients with unsuccessful ablation (12.7% ± 6.9% vs. 4.9% ± 4.1%, *p* = 0.006), and preoperative LA strain was a statistically significant predictor for freedom from AF in logistic regression analysis. Furthermore, we have shown the high efficacy and technical feasibility of LA cryoablation with concomitant LAA clipping in patients undergoing minimally invasive mitral valve surgery with a rate of freedom from AF of 72% (68% off AAD) after 1-year follow-up.

Concomitant surgical AF ablation is an established therapy in patients with AF undergoing cardiac surgery (10). In patients with persistent and especially long-standing persistent AF, many surgeons are reluctant to perform a surgical ablation, due to various reasons. On the one hand, the lack of training might be an issue. On the other hand, when not using proper ablation techniques, the success rates that can be achieved are rather low. In addition to the reduction of stroke risk, the restoration of sinus rhythm by AF ablation can contribute to the restoration of atrial contractility and atrial transport function. LA function decreases with the duration of AF. In addition to the LA dilatation, atrial fibrosis contributes to reduced atrial contractility (19). For several years, LA diameter and LA volume were used as echocardiographic parameters to characterize the left atrium. However, the LA function is more precisely characterized by LA strain. LA strain can be easily obtained in transthoracic echocardiography and can be used to adequately characterize LA function. In recent studies of catheter ablation, LA strain has already been used as a rhythm outcome predictor (18, 23). Furthermore, in a study incorporating patients with long-standing persistent AF undergoing thoracoscopic surgical ablation or catheter ablation, LA strain at 3 months postoperative was a predictor for successful ablation (24).

According to a study by Boano et al. (25), the effects of surgical cryo- and radiofrequency ablation concomitant to mitral valve surgery on LA function were assessed. Here, the authors did not find any differences in preoperative LA strain in patients with successful and unsuccessful ablation.

According to a study by van Kampen et al. (26) incorporating 251 patients, after surgical mitral valve repair, LA strain was shown to be a predictor for mid-to-late onset of AF. Including LA strain in surgical decision-making may identify patients who will benefit from earlier intervention to prevent irreversible LA damage with the risk of postoperative AF.

Since LA strain can detect changes in LA function at an early stage, possibly in patients with severe mitral regurgitation, it can be used as an indicator for hemodynamic consequences of the mitral regurgitation before overt LA structural changes are present and can be helpful to determine timepoint of operation in the future. Unfortunately, to date, there are no cutoff values for the timing of mitral valve surgery available, and further research is needed in that field.

Here, we were able to demonstrate that LA strain is also a predictor for successful rhythm restoration after surgical ablation. In our study, all patients were treated with the same LA lesion set and received LAA occlusion using the AtriClip. Due to the same AF treatment in all patients, the factor of different energy sources or lesion sets influencing rhythm results can be excluded. In contrast to LA strain, LA volume was not associated with the rhythm outcome. Therefore, in our study, LA strain had an additive value over LA volume and can more adequately predict the rhythm outcome in this patient cohort.

A biatrial ablation is the preferred method by many AF surgeons, especially in patients with persistent or long-standing persistent AF. However, there is an ongoing discussion about when to use a biatrial and when an LA lesion is set. Some previously published studies have reported superior outcomes for patients treated with a biatrial lesion set in comparison to an LA ablation. A meta-analysis by Barnett et al. (6) including 5,885 patients undergoing concomitant surgical ablation found that patients receiving a biatrial lesion set had statistically higher rates of freedom from AF compared to those receiving an LA lesion set only. Similar results were shown in a meta-analysis by McClure et al. (10). However, there are also conflicting results, where no difference in the rhythm outcome between both methods was described. A study by Soni et al. (27) showed the rates of freedom from AF in patients who underwent biatrial lesion set, LA lesion set, and pulmonary vein isolation were 80.0%, 76.1%, and 56.9%, respectively, at 12 months of follow-up. This analysis suggested no improvement in patients who underwent an additional right atrial lesion set. Similar results were shown in a meta-analysis by Li et al. (28). Moreover, in some previously published studies, patients treated with a biatrial ablation had a higher rate of permanent pacemaker implantation compared to those receiving LA lesions only (29–31). Furthermore, a prospective randomized study by Gillinov et al. (17) was not able to show the benefit of a biatrial lesion set in comparison to PVI only in patients undergoing surgical ablation concomitant to mitral valve surgery. Therefore, many surgeons prefer an LA lesion set only, especially when using minimally invasive- or robotic access. However, one can imagine the more diseased the left atrium is, the higher the probability that this atrial disease is not only restricted to the left atrium but also to the right atrium. Here, an additional right atrial ablation might be beneficial. Since it is difficult to predict which patient benefits from additional right-sided lesions, in the future, preoperative LA strain might be used to guide decision-making. In patients with less diseased left atria, an LA lesion set might be sufficient, while patients with low LA strain values might benefit from a biatrial lesion set. However, this hypothesis needs to be tested in further prospective, randomized studies.

Our results of freedom from AF of 72% after 1-year follow-up are quite encouraging in this group of patients with persistent and long-standing persistent AF. These results are in line with other previously published studies, although these studies included patients with paroxysmal and persistent AF (27). Furthermore, we have confirmed the technical feasibility of LAA closure using the AtriClip through a right lateral mini-thoracotomy in a larger patient cohort. We have shown successful intraoperative LAA closure in all patients, illustrating the simplicity and reproducibility of this LAA occlusion technique through the transverse sinus. The importance of LAA closure concomitant to cardiac surgery has been shown in LAAOS III, a randomized controlled trial, where patients in the concomitant LAA closure group had a 33% reduced stroke and arterial embolism rate in comparison to patients receiving no LAA closure (32). Furthermore, it has been shown in previously published studies that the LAA closure technique is very important and several techniques lead to an unsuccessful or incomplete LAA closure (33).

## Conclusion

In this study, we have shown that preoperative LA strain analysis is a valuable tool for predicting the rhythm outcome in patients with persistent and long-standing persistent AF undergoing concomitant surgical ablation. In the future, LA strain might be used as a tool to guide decision-making on ablation lesion set in patients with persistent and long-standing persistent AF.

### Limitations

This study is a retrospective study with the potential risk of bias by unknown confounders. Furthermore, our series is a single-center study and is limited by the heterogeneity of our patient population and the relatively small patient number. In addition, the follow-up is limited to 12 months.

## Data Availability

The raw data supporting the conclusions of this article will be made available by the authors, without undue reservation.
